# Accounting for the relationship between per diem cost and LOS when estimating hospitalization costs

**DOI:** 10.1186/1472-6963-12-439

**Published:** 2012-12-01

**Authors:** K Jack Ishak, Marilyn Stolar, Ming-yi Hu, Piedad Alvarez, Yamei Wang, Denis Getsios, Gregory C Williams

**Affiliations:** 1 United BioSource Corporation, 185 Dorval Ave., Suite 500, Dorval, QC, H9S 5J9, Canada; 2 United BioSource Corporation, 230 Bedford Street, Suite 300, Lexington, MA, 02420, USA; 3 The Medicines Company, 8 Sylvan Way, Parsippany, NJ, 07054, USA

## Abstract

**Background:**

Hospitalization costs in clinical trials are typically derived by multiplying the length of stay (LOS) by an average per-diem (PD) cost from external sources. This assumes that PD costs are independent of LOS. Resource utilization in early days of the stay is usually more intense, however, and thus, the PD cost for a short hospitalization may be higher than for longer stays. The shape of this relationship is unlikely to be linear, as PD costs would be expected to gradually plateau. This paper describes how to model the relationship between PD cost and LOS using flexible statistical modelling techniques.

**Methods:**

An example based on a clinical study of clevidipine for the treatment of peri-operative hypertension during hospitalizations for cardiac surgery is used to illustrate how inferences about cost-savings associated with good blood pressure (BP) control during the stay can be affected by the approach used to derive hospitalization costs.

Data on the cost and LOS of hospitalizations for coronary artery bypass grafting (CABG) from the Massachusetts Acute Hospital Case Mix Database (the MA Case Mix Database) were analyzed to link LOS to PD cost, factoring in complications that may have occurred during the hospitalization or post-discharge. The shape of the relationship between LOS and PD costs in the MA Case Mix was explored graphically in a regression framework. A series of statistical models including those based on simple logarithmic transformation of LOS to more flexible models using LOcally wEighted Scatterplot Smoothing (LOESS) techniques were considered. A final model was selected, using simplicity and parsimony as guiding principles in addition traditional fit statistics (like Akaike’s Information Criterion, or AIC). This mapping was applied in ECLIPSE to predict an LOS-specific PD cost, and then a total cost of hospitalization. These were then compared for patients who had good vs. poor peri-operative blood-pressure control.

**Results:**

The MA Case Mix dataset included data from over 10,000 patients. Visual inspection of PD vs. LOS revealed a non-linear relationship. A logarithmic model and a series of LOESS and piecewise-linear models with varying connection points were tested. The logarithmic model was ultimately favoured for its fit and simplicity. Using this mapping in the ECLIPSE trials, we found that good peri-operative BP control was associated with a cost savings of $5,366 when costs were derived using the mapping, compared with savings of $7,666 obtained using the traditional approach of calculating the cost.

**Conclusions:**

PD costs vary systematically with LOS, with short stays being associated with high PD costs that drop gradually and level off. The shape of the relationship may differ in other settings. It is important to assess this and model the observed pattern, as this may have an impact on conclusions based on derived hospitalization costs.

## Background

Data from clinical trials are often used to support economic evaluations or to perform post-hoc analyses of health-economic outcomes (e.g., difference in total cost of care between interventions). Trials that are not designed to capture economic data (i.e., charges for services and treatments provided during the stay) lack the detail needed to derive the exact cost of the hospitalizations. These must, therefore, be approximated using other information about the hospitalization such as the length of stay (LOS) multiplied by a per diem (PD) cost. The PD cost must be obtained from publications or derived from supplemental data sources where the total cost and LOS of similar hospitalization are recorded. An alternative (but cruder) approach to obtain an average PD cost is to divide an institution’s total costs for a particular period by the total patient-days [1-4].

Using an average PD cost may result in a biased estimate of the cost of the event if other factors that may influence the PD costs are not considered [5,6]. These factors include the reason for hospitalization, the severity of the patient’s condition, and presence of comorbidities. PD costs can be made more specific by restricting the calculation to a particular case mix [3,7], or deriving patient-specific (e.g., by disease severity), disease-specific [2] or ward-specific [8,9] PD costs. This may not be sufficient, however, since PD costs are also closely associated with LOS. [10,11] It has been shown that while the total cost of hospitalization may increase with LOS, the average PD cost generally decreases as the LOS increases [12,13], since the most of the costs accrue immediately after admission or as complications arise, and drop substantially during the recovery phase of the hospital stay. Using a PD cost that is not adjusted for LOS can lead to inaccurate total cost estimates, and distort comparisons of costs between groups, particularly when LOS differs between the groups.

Adjusting for LOS in the derivation of PD costs is not necessarily straight-forward due to the potentially complex (e.g., non-linear) relationship between the variables. One approach to dealing with this may be to categorize LOS into intervals, and calculate average PD cost within each of these. The optimal number of intervals and cutoff points may be difficult to determine and small counts within some intervals can be limiting. In this paper, we describe an approach based on flexible statistical modeling techniques to predict an LOS-specific PD cost. The method is described and illustrated with the analyses of hospitalization costs in a clinical study of clevidipine for treatment of perioperative (pre-, intra- and post-operative) hypertension in the setting of cardiac surgery. The potential impact of the approach to derive PD costs on the association between blood pressure (BP) control and total cost is illustrated.

## Methods

### Case study: is better peri-operative blood pressure control associated with lower cost of hospitalization for cardiac surgery?

The ECLIPSE [14] (Evaluation of CLevidipine In the Perioperative Treatment of Hypertension Assessing Safety Events) Trials compared the safety and efficacy of clevidipine with nitroglycerin, sodium nitroprusside, and nicardipine in the treatment of perioperative hypertension in patients undergoing coronary artery bypass grafting (CABG), valve surgery or combined surgeries. ECLIPSE included three parallel, randomized, open-label studies conducted at 61 medical centers in the United States between April 2004 and October 2006.

Efficacy was assessed by degree of control of systolic BP measured by the portion of the area under the curve (AUC) of systolic BP over time that fell outside (either above or below) of the range defining control (75–145 mm Hg intra-operatively and 85–155 mm Hg pre- and postoperatively) during the 24-hour period following study drug initiation. The AUC values were normalized per hour and expressed in units of mmHg × min/h; larger AUC indicated a lesser degree of BP control, and, hence, greater BP variability [15]. An analysis of the pooled populations of the trials (N = 1,512) showed that better BP control by decreasing peri-operative systolic BP variability was associated with a significant reduction of 30-day mortality [16]. Data from the trial alone did not allow examination of whether better BP control was also associated with lower total cost for the surgery because medical charges were not recorded during the trial. Only the LOS of the index hospitalization (i.e., for CABG, valve replacement or combination surgery) and occurrences of pre- and post-discharge complications were available. Thus, supplemental data were required to derive pre- and post-discharge costs, and examine the association between the peri-operative BP control and the total cost.

### Derivation of total costs

#### Data requirements

Total cost was defined as the sum of the cost of the index hospitalization, and any costs incurred post-discharge due to complications. The former may be derived based on the LOS of the hospitalization and PD costs that take into account the type of surgery performed and any complications that may have occurred, as these may significantly impact the cost. As no details are available for post-discharge events, a mean cost for each type of complication is required.

Complications expected to have an important influence [15,17,18] on both pre- and post-discharge costs were: myocardial infarction (MI), stroke, infection, renal failure, bleeding event(s), and death. These complications were selected due to their high costs and potential for providing detectable cost differences in comparisons between patients with good vs. poor peri-operative BP control. This was based on clinical considerations; different complications have different courses of treatment and recovery, and thus different patterns of cost and LOS.

The following complication groupings were defined. The data would not permit analyses for cases where multiple complications occurred; thus, these cases were grouped as having two complications or more than two complications.

1. Death (with or without other complication)

2. MI only

3. Stroke only

4. Infection only

5. Renal failure only

6. Bleeding event only

7. Two complications

8. More than two complications

9. Other (i.e., none of the above, including possibly no complications)

Patients were classified into one of these groups based on their experience during the index hospitalization. In addition to the complications, the index hospitalization cost was also expected to depend on the type of surgery. Thus, PD costs were required for 27 different scenarios (3 types of surgery x 9 complication groups). To further account for a potential association between PD costs and LOS, individual-level data were required to derive a suitable mapping. As no details were available for post-discharge complications, a mean cost for hospitalizations for each type of specific complication (i.e., death, MI, stroke, infection, renal failure and bleeding event) were also required to be derived post-discharge cost for patients in ECLIPSE.

#### Cost data source

Data from the Massachusetts Acute Hospital Case Mix Database (henceforth, the MA Case Mix Database) were used to obtain pertinent cost information to derive pre- and post-discharge costs for patients in the ECLIPSE trial. The MA Case Mix database includes data on charges, LOS, diagnoses and procedures, as well as socio-demographic information for patients of all ages covered by all payers. This database has been used previously to evaluate hospitalization costs and LOS for CABG surgery including post-operative complications [19,20].

To ensure compatibility with the ECLIPSE trials, the database was restricted to the 2005–2007 [17] period, and identified patients with a hospitalization for a CABG (ICD-9-CM procedure codes 36.10–36.19) or Valve Surgery (35.10–35.28, 35.31, 35.32, 35.33, 35.99) or both, with appropriate DRG codes (N = 18,548). Each hospitalization was classified into one of the nine complication groups based on concurrent diagnoses reported in the hospitalization record (see Additional file 1 for specific ICD-90-CM codes used). The cost for each hospitalization was calculated using the average Massachusetts cost to charge ratios from Healthcare Utilization Project (HCUP) National Inpatient Sample files specific for each year of the data (2005 – 2008) [21]: 0.559084 for 2005, 0.559934 for 2006, and 0.570321 for 2007. The costs were then inflated to 2009 dollars using the Bureau of Labor Statistics inflation rates for New England [22]: 1.201 for 2005, 1.156 for 2006, and 1.096 for 2007. Finally a PD cost for each hospitalization was calculated by dividing the cost of the hospitalization by its LOS.

This produced a dataset including the following variables: type of cardiac hospitalization, complication group, LOS and PD Cost, as well as basic demographic information on patients – i.e., age, sex and race (white, non-white). To ensure compatibility with the ECLIPSE data, the distribution of LOS values in each of the complication groups were compared with those in ECLIPSE. Hospitalizations with LOS falling outside of the range (minimum- maximum) observed in ECLIPSE were excluded. A total of 158 (1.5%) observations were dropped, as these might reflect types of patients that are not representative of the ECLIPSE populations. For instance, cases with very long LOS may have concomitant chronic conditions that account for their very late discharge, while cases with very short LOS may be indicative of a transfer to other facility rather than a true discharge. The resulting dataset, which included 10,450 observations was used to examine the relationship between PD cost and LOS, as described below.

Mean costs for post-discharge complications were also derived from the MA Case Mix Database restricted to the 2005–2007 period. Hospitalizations with a principal diagnosis corresponding to each type of complication (Appendix A) were identified. For patients with CABG, the records were restricted to those with a secondary diagnosis indicating Aortocoronary Bypass Status (code V45.81). All charges were adjusted by 0.570321, the mean cost-to-charge ratio from the 2007 HCUP Nationwide Inpatient Survey Cost-to-Charge Ratio Files - Massachusetts. These were then inflated to 2009 dollars by applying an adjustment factor of 1.096 [23]. This yielded a dataset including post-discharge and complication group. The mean cost for each type of complication was derived.

#### Statistical analysis: derivation of appropriate PD cost for ECLIPSE patients

The traditional approach to deriving a PD Cost would involve calculating a mean value of observations in the MA Case Mix dataset for each of the 27 surgery/complication scenarios defined above. These would then be multiplied by the LOS of patients in corresponding surgery/complication groups to obtain the cost of the index hospitalization. This approach assumes that within each of the 27 subcategories, PD cost and LOS are independent, or equivalently, that the hospitalization cost is proportional to LOS.

The validity of this assumption should be examined using graphical displays, and regression techniques, particularly models that allow flexible shapes, to explore the relationship between PD cost and LOS. Since costs are known to have a skewed distribution [21,24] and are non-negative (i.e., > 0), the usual linear regression models cannot be used, as these assume a normal distributions for the dependent variable. Other models based on log-normal or gamma distributions are often used. [21] Alternatively, a natural log transformation of costs is also possible. We adopted the latter approach to have the greatest flexibility in exploring several modelling approaches.

The first step involved exploratory analyses to help identify an appropriate modelling approach. There were two main considerations:

1. What statistical techniques can best capture the observed shape of the relationship between PD cost and LOS, and

2. Whether the nine complication groups can be analyzed jointly with a single model including LOS and complication type as predictors and possible interaction terms, or whether this is more appropriately done with separate models for each of the groups.

Scatter plots of PD costs vs. LOS were created for the nine groups. A visual examination of these graphs provided clues about an appropriate shape. This was aided further by adding LOESS (LOcally wEighted Scatterplot Smoothing) curves to the plots. A LOESS curve is derived by fitting a simple model to localized subsets of the data to build up a function that describes the deterministic part of the variation in the data, point by point. By overlaying this function on the scatterplot, the trend and noise can be visually separated. One of the main features of this method is that the data analyst is not required to specify a global function of any form to fit a model to the data, only to fit segments of the data [23,25]. Based on the shape of the derived function, however, candidate parametric functions (e.g., linear, quadratic, logarithmic, etc.) may be identified for formal testing. If a simple functional form does not seem adequate, piecewise functional forms should be considered. For instance, piecewise linear models can be used with knots set at appropriate points to capture changes in shape.

The parametric functions identified for log of PD costs vs. LOS are described in the results section. These were fitted to the observed data to assess their fit and determine a final optimal model using the Akaike information criterion (AIC) [26] and Bayesian information criterion (BIC) [27] statistics, as well as examination of observed vs. predicted plots. Parsimony and simplicity were used as deciding factors to choose between models with comparable fit.

Since the aim of the paper is to illustrate the application of this approach, the details of the process of determining candidate models and choosing a final mapping are described in the results section.

#### Application in ECLIPSE to compare total cost for patients with poor vs. good BP control

**Figure 1:**
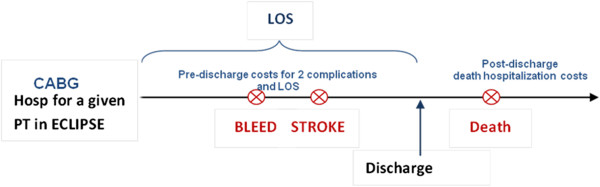
Classification of patients in ECLIPSE to derive total cost based on pre- and post-discharge events.

The mapping between LOS and PD Costs derived in the previous step was used to derive the cost of the index hospitalization by multiplying the predicted PD cost by the LOS observed in ECLIPSE. We also derived the pre-discharge cost based on an average PD cost that ignores the relationship with LOS to illustrate the potential impact on conclusions about BP control and total cost. A post-discharge cost was derived based on complications occurring after the index hospitalization. For patients with multiple post-discharge complications, the cost of the most expensive event was used. The pre- and post-discharge costs were added to obtain the total cost for each patient in ECLIPSE. Figure 1 illustrates an example of a patient who had a bleed followed by a stroke during the index hospitalization and died after discharge. The PD cost for the Two Complications group would be use to derive the pre-discharge cost based on the LOS, and a post-discharge cost would be based on a hospitalization for death.

The following post-discharge cost values were used for the patients with CABG procedure only: Infection: $2,090.63; Bleed: $8,859.85; Renal: $10,067.23; Stroke: $14,787.09; Renal death: $15,221.99; Stroke death: $16,087.93; other death: $18,284.28; MI; $18,937.24. The corresponding cost was added to the pre-discharge cost to obtain the total cost.

The mean total cost of patients with good vs. poor BP control in ECLIPSE was compared. Good systolic BP control was defined as AUC below 10 mm Hg × min/h, and values above were considered indicative of poor control. The 10 mm Hg × min/h cutoff was identified in exploratory analyses examining variations in costs in deciles of the AUC variable. This was done with total costs calculated with the LOS-specific and mean PD costs (i.e., the traditional crude approach) to illustrate the potential impact of capturing the actual shape of the PD cost vs. LOS association.

## Results

Patients in ECLIPSE may have undergone coronary artery bypass grafting (CABG), valve surgery or combined surgeries. Analyses were performed separately for each type of surgery. For brevity, results are reported for analyses for the group of 1,165 ECLIPSE patients who had only a CABG procedure.

### Analysis population from the MA case mix database

Over 10,000 patients undergoing a CABG procedure were identified from the MA Case Mix Database between year 2005 and 2007. Table 1 shows the distribution of patients across complication groups, along with a basic demographic characterization of the populations in the MA Case Mix and ECLIPSE.

**Table 1:** Distribution of Complications and Basic Demographic Profile of MA Case Mix Population and ECLIPSE Trials for CABG Only Cohorts

**ALL**	**ECLIPSE**				**MA Case Mix Database**			
	**N (%)**	**Sex (% Male)**	**Age Mean (SD)**	**Race (% Not White)**	**N (%)**	**Sex (% Male)**	**Age Mean (SD)**	**Race (% Not White)**
	**1,164**	**76.5**	**64.4 (10.1)**	**17.3**	**10,450**	**76.0**	**66.4 (10.8)**	**16.1**
Death	26 (2.2)	65.4	70.0 (9.7)	23.1	134 (1.3)	59.0	73.1 (11.0)	17.2
Bleed(s)	96 (8.2)	74.0	66.7 (9.3)	24.0	561 (5.4)	77.0	68.7 (11.0)	13.7
MI	11 (0.9)	45.5	61.5 (6.4)	0.0	116 (1.1)	70.7	66.0 (11.1)	15.5
None/Other	965 (82.9)	77.2	63.8 (10.2)	15.8	8,624 (82.5)	76.7	65.8 (10.7)	16.0
Infection	3 (0.3)	66.7	49.3 (7.8)	0.0	204 (2.0)	72.6	66.9 (10.3)	14.7
>2 Complications	5 (0.4)	60.0	66.6 (11.3)	40.0	26 (0.3)	92.3	71.8 (9.3)	34.6
Stroke	3 (0.3)	100.0	66.7 (7.6)	33.3	110 (1.1)	67.3	69.4 (10.3)	20.0
2 Complications	21 (1.8)	81.0	72.5 (7.6)	23.8	212 (2.0)	71.2	69.7 (10.7)	21.7
Renal	34 (2.9)	79.4	64.7 (9.0)	32.3	463 (4.4)	73.2	70.7 (10.0)	17.7

* Complications sorted in order of increasing PD cost.

The majority of patients in both populations are predominantly in the group with none/other complications (over 80%); very few patients had more than 2 complications (less than 0.5%). Complications related to bleeds were observed more frequently in the ECLIPSE trials (8.2% vs. 5.4%); infections (0.3% vs. 2.0%) and strokes (0.3% vs. 1.1%) occurred less frequently in ECLIPSE. Deaths were less common among MA cases (1.3% vs. 2.2%). The demographic profile of patients is generally similar, although some variation is apparent within some complication groups (e.g., infection, > 2 complication groups). It should be noted, that these groups included very few patients (e.g., three patients with infection, five patients with > 2 complications). Thus, we concluded that the MA database is adequately representative of the ECLIPSE patients.

The LOS distributions for MA cases and ECLIPSE patients are presented in Table 2. Apparent LOS outliers that far exceeded the range of values observed in ECLIPSE (158 cases, 1.5%) were excluded. The distributions of PD costs by complication group are also shown in Table 2. Large SDs of PD costs (relative to the means), and the distance between means and medians indicate the right skew of the cost distributions within complication groups. Mean PD costs range from approximately $5,000 for renal complications or stroke to $6,500 for bleeds. Death for any cause is the most costly post-operative event but highly variable. The higher PD costs for the bleeds and none/other groups may be partly explained by the shorter lengths of stay (approximately 8–10 days compared with 15 days for patients with renal failure) and is indicative of a non-linear relationship between PD costs and LOS.

**Table 2:** Distribution of LOS in the MA Case Mix Population and in ECLIPSE

**Complication Group**	**ECLIPSE**			**MA Case Mix Database**				
	**LOS (Days)**				**LOS (Days)**		**PD Costs ($)**	
	**N (%)**	**Mean (SD)**	**Min-Med-Max**	N* (%)	**Mean (SD)**	**Min-Med-Max**	**Mean (SD)**	**Min-Med-Max**
ALL	1,164	8.3 (5.9)	1 - 7 - 74	10,450	9.3 (5.1)	1 - 8 - 58	--	--
Death	26 (2.2)	11.9 (10.4)	1 - 7.5 - 32	134 (1.3)	11.1 (8.2)	1 - 8 - 32	15,434 (14,562)	2,958 – 10,997 – 89,957
Bleed(s)	96 (8.2)	8.9 (5.1)	4 - 8 - 46	561 (5.4)	10.4 (5.4)	4 - 9 - 43	6,503 (2,581)	1,851 – 6,162 – 17,762
MI	11 (0.9)	10.5 (3.8)	6 - 11 - 17	116 (1.1)	10.1 (3.1)	6 - 10 - 17	6,353 (2,726)	2,061 – 5,828 – 14,375
None/Other	965 (82.9)	7.4 (4.1)	3 - 7 - 74	8,624 (82.5)	8.4 (4.1)	3 - 7 - 58	6,188 (2,288)	30 – 5,883 – 26,193
Infection	3 (0.3)	14 (9.2)	6 - 12 - 24	204 (2.0)	12.2 (4.6)	6 - 12 - 24	5,968 (2,150)	2,040 – 5,914 – 12,062
>2 Compls	5 (0.4)	25.2 (13.9)	7 - 22 - 41	26 (0.2)	25.6 (8)	10 - 26 - 41	5,804 (1,842)	3,009 – 5,333 – 9,753
Stroke	3 (0.3)	24 (23.4)	9 - 12 - 51	110 (1.1)	15.1 (5.9)	9 - 13 - 46	4,929 (1,730)	1,825 – 4,587 – 10,773
2 Compls	21 (1.8)	24.9 (18)	6 - 17 - 62	212 (2.0)	18.8 (9.1)	6 - 17 - 57	5,208 (2,156)	1,842 – 4,881 – 15,161
Renal	34 (2.9)	11.3 (7.4)	6 - 0 - 42	463 (4.4)	15 (6.9)	6 - 14 - 42	5,035 (1,895)	1,802 – 4,798 – 13,252

* 158 (1.5%) observations were excluded to match the range of LOS in ECLIPSE.

Min = Minimum; Med = Median; Max = Maximum.

### Exploring the relationship between PD cost and LOS in the MA database

**Figure 2:**
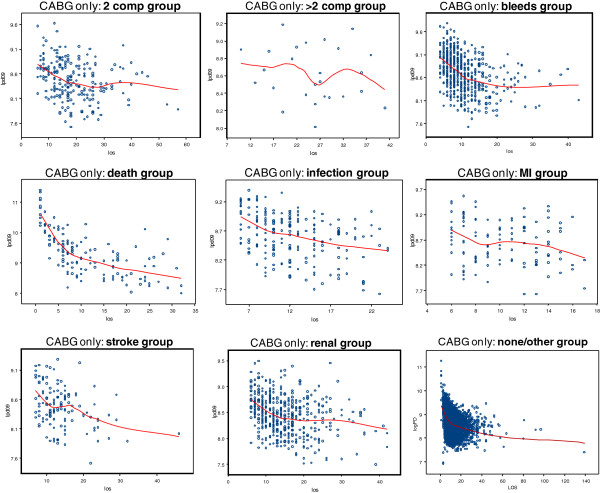
LOESS Plots for LnPDcost vs. LOS for 9 Complication Groups in the MA Case Mix Database.

Figure 2 shows the scatter plots of the Ln of PD costs versus LOS for the nine complication groups, along with LOESS curves. In each case, the PD cost is higher for very short LOS compared with PD costs at the longer LOS values. The shapes tend to curve sharply downward up to LOS of 14 days, and then continue to curve downward at a slower rate thereafter. Although some deviation from this pattern are seen where data is sparse, the plots reveal a generally similar pattern in the nine groups. Thus, a single model was deemed appropriate to capture the relationship in all of the groups using interaction terms between the LOS variable and group indicators to allow flexibility in the shape of the association across complication types. Thus, the modelled shape for the smaller groups borrow strength from the larger ones, while still allowing the shapes observed in the underlying data to exert influence.

The shape implied by the LOESS curves can be parameterized in different ways. A piecewise model could be used to capture the initial sharp decline up to day 14 and the subsequent slower decline or flattened pattern. A few variations are possible: the segments in the piecewise model may be best captured by linear equations (i.e., the overall model would consist of two straight lines connected at the knot), or a more complex form like a quadratic equation for each segment may provide better fit. The scatter plots leave some doubt as to whether the shape of the relationship changes after day 30; thus, a second knot could be added at this point. Alternatively, it may be that variability noted after day 14 is spurious and due to the sparseness of data; therefore, the true model may be that for hospitalizations lasting two weeks or longer, the PD cost is relatively constant. Thus, a flat line may work best as a second segment in the overall model. Finally, a logarithmic model (i.e., log of PD Cost vs. log of LOS) may capture the observed shape equally well, as the observed pattern resembles the functional form of the log function. Based on these considerations, the following models were fitted and compared:

● Piecewise linear with knot at 14 days and knot at 30 days

● Piecewise linear with knot at 14 days

● Piecewise linear with knot at 14 days, horizontal after 14 days

● Piecewise quadratic with knot at 14 days

● Piecewise quadratic with knot at 14 days, horizontal after 14 days

● Logarithmic

A piecewise quadratic function with knots at days 14 and 30 was not considered due to the greater complexity of the model relative to the available data points past day 30.

### Model fitting and selection

**Figure 3:**
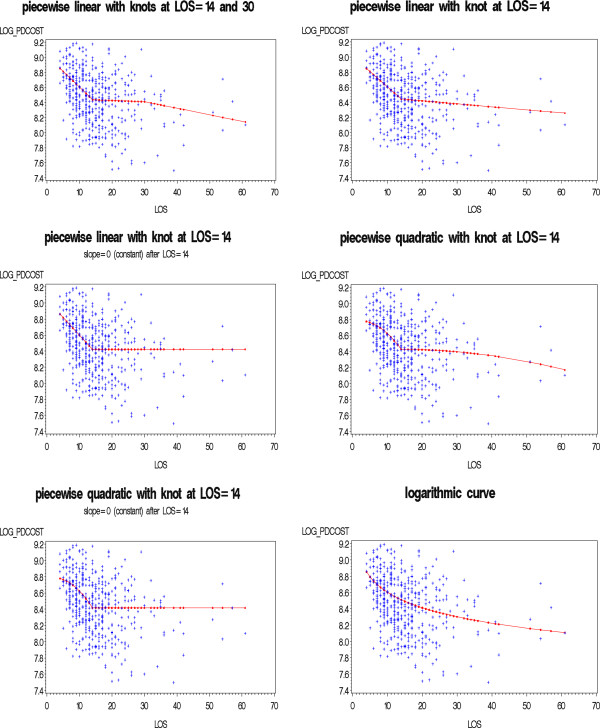
Candidate Models for the CABG-only with Renal Complication Group in the MA Case Mix Database.

Each of the six candidate models was fitted to the MA data. The models included an intercept, indicators for complication groups, appropriate terms for the parameterization of LOS, and an interaction between the latter and complication groups. For instance, in one model, LOS was log-transformed; thus, the interaction terms with complication group allowed the coefficient for Ln LOS to change, building in a change in shape. For comparison, an intercept-only (or horizontal line) model was also fitted, as this reflects the relationship implied by the traditional approach. Figure 3 illustrates each of the candidate models using the fitted curves and the underlying data for the renal complication group.

The goodness-of-fit statistics (AIC and BIC) for each of the six candidate models along with the traditional model assuming constant PD cost for all LOS are summarized in Table 3. The traditional model had the poorest fit (i.e., largest AIC and BIC values), while the piecewise linear and logarithmic models had the better fits. The piecewise quadratic models were not considered further, as they had the largest BIC values; thus, the added complexity of the model did not contribute to a better fit. Similarly, adding a knot on day 30 led to no improvement in fit; in fact, the BIC statistic for this model suggests considerable loss in fit. Thus, the final choice was between the piecewise linear and logarithmic models (model C and F in Table 3), which had comparable fit, but favoring its simplicity and smoothness, we opted for the logarithmic model.

**Table 3:** Fit Statistics for Candidate Models (MA claims)

**Model**	**Fit Statistics**	
	**AIC**	**BIC**
**(A) Piecewise linear with knots at 14 and 30 days**	7,666.8	7,920.6
**(B) Piecewise linear with a knot at 14 days**	7,664.9	7,868.0
**(C) Piecewise linear with a knot at 14 days and horizontal tail**	7,665.3	7,803.1
**(D) Piecewise quadratic with a knot at 14 days**	7,626.9	7,960.6
**(E) Piecewise quadratic with a knot at 14 days and horizontal tail**	7,621.4	7,824.5
**(F) Logarithmic**	7,666.0	7,803.8
**(G) Constant (Traditional)**	9,605.0	9,677.6

AIC: Akaike Information Criterion BIC: Bayesian Information Criterion.

**Figure 4:**
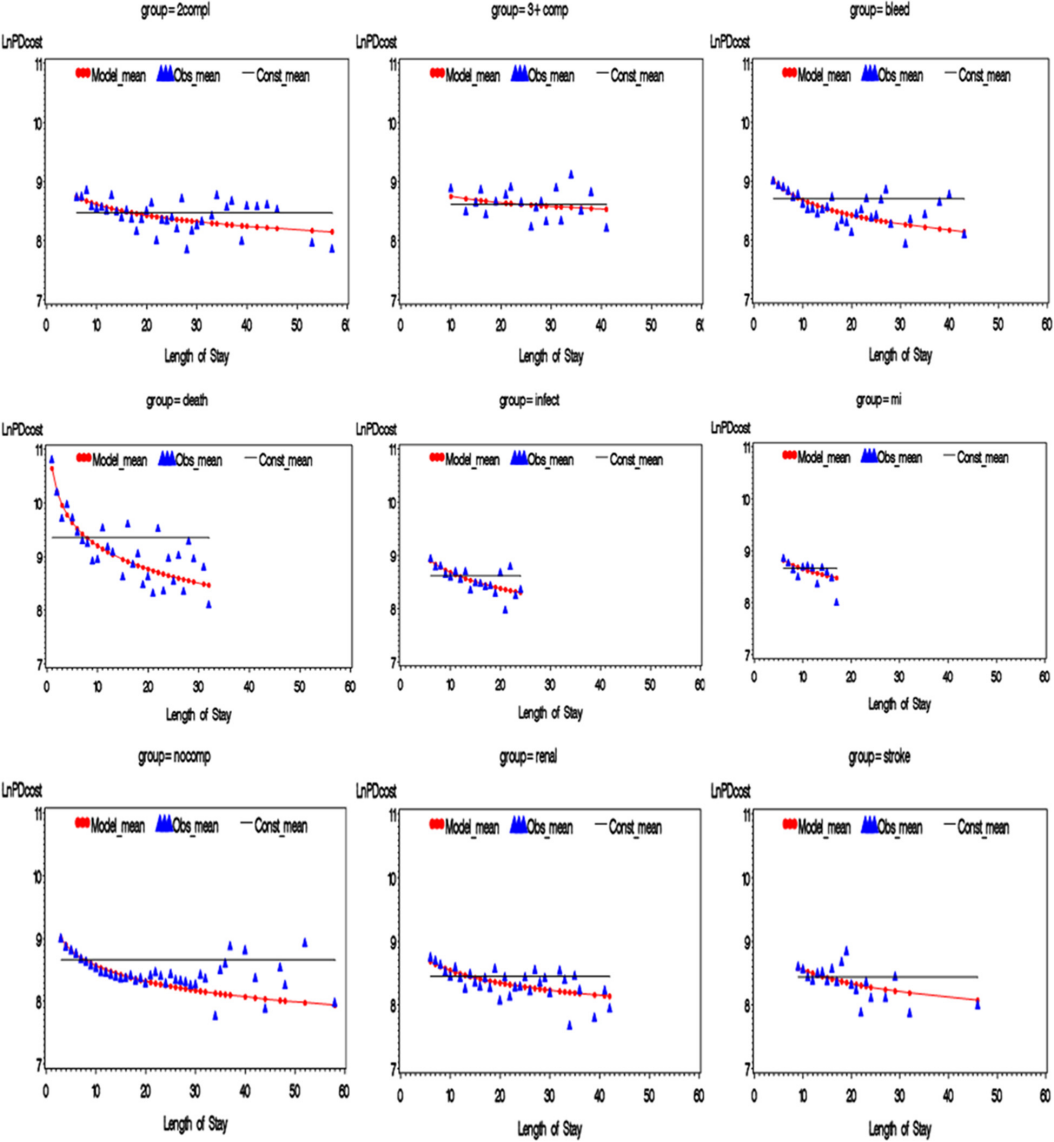
Predicted Means from Logarithmic and Constant Models with Observed Means Ln PD cost versus LOS by Complication Group in the MA Case Mix Database.

The fitted final model is summarized in Table 4. The coefficients represent a change in Ln PD cost for a unit increase in the predictor. Predicted values from the constant and logarithmic models are plotted in Figure 4, overlaid with the mean of the observed Ln PD cost for each LOS in the MA database for each complication group.

**Table 4:** Logarithmic and Constant Models for Ln PD Cost (MA Database)

		**Logarithmic Model (F)**		**Constant Model (G)**	
		**Estimate (SE)**	**P-Value**	**Estimate (SE)**	**P-Value**
Intercept		9.281 (0.271)	<.0001	8.445 (0.037)	<.0001
Ln(LOS)		−0.315 (0.101)	0.002	NA	NA
Complication Group	Death	1.365 (0.281)	<.0001	0.903 (0.049)	<.0001
	Bleed	0.25 (0.281)	0.375	0.258 (0.04)	<.0001
	MI	0.107 (0.362)	0.769	0.219 (0.051)	<.0001
	None/Other	0.110 (0.271)	0.685	0.218 (0.037)	<.0001
	Infection	0.384 (0.315)	0.224	0.179 (0.045)	<.0001
	>2 Compls	−0.17 (0.699)	0.808	0.173 (0.083)	0.038
	2 Compls	−0.053 (0.307)	0.864	0.034 (0.045)	0.444
	Renal	−0.086 (0.288)	0.765	0.013 (0.041)	0.759
	Stroke	Reference*		Reference*	
Ln(LOS) x Compl. Group	Death	−0.313 (0.106)	0.003		
	Bleed	−0.054 (0.107)	0.611		
	MI	−0.005 (0.146)	0.973		
	None/Other	−0.042 (0.102)	0.677		
	Infection	−0.113 (0.121)	0.35		
	>2 Compls	0.16 (0.225)	0.477		
	2 Compls	0.049 (0.113)	0.662		
	Renal	0.032 (0.108)	0.765		
	Stroke	Reference*			

* Patients with stroke are used as the reference category for indicators of complication: coefficients represent difference in log PD costs for each type of complication relative to stroke.

### Application: comparison of total hospitalization cost for patient with poor vs. good BP control

**Figure 5:**
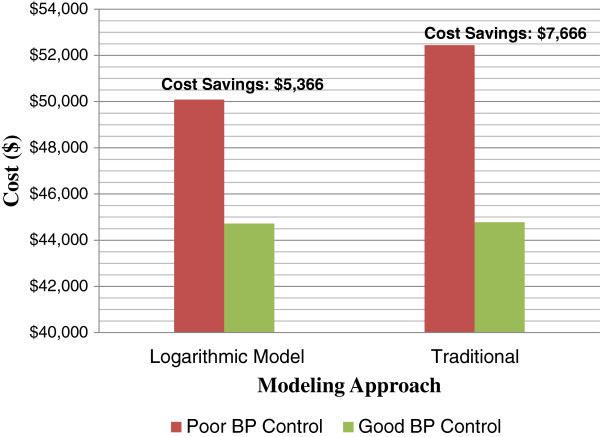
Estimated Total Cost Savings Comparison for Model-based and Traditional Methods.

We used the final logarithmic model and the traditional (constant) model fitted to each complication group to calculate PD costs for patients in the ECLIPSE trials and added post-discharge costs to obtain the total pre- and post-hospitalization cost. The mean total costs for patients with poor vs. good perioperative BP control for patients in the CABG group are summarized in Figure 5. Estimated costs with the crude and modeled approach were similar for patients with good BP control, but differed by over $2,000 for the poor BP control group. This leads to a 43% overestimation of the cost savings associated with good BP control.

## Discussion

Derivation of hospitalization costs is a common requirement in health economic modeling exercises, as these are often not available in the primary data source being used as the basis for the model. If suitable external data can be identified with both costs and LOS, the approach described in this paper can be applied to improve the accuracy of calculations by explicitly modeling the relationship between PD costs and LOS. In addition to LOS, other factors, such as types of complications occurring during the hospitalization can also be useful, as long as these are captured in both sources of data.

Modeling the relationship between PD costs and LOS is likely to require consideration of methods that can accommodate non-linear functional forms. Non-linear functions (e.g., logarithmic) and piecewise modeling are two possible approaches. Other methods, such as spline models [28] and fractional polynomial models [29] may also work well. Parsimony and simplicity should be considered as guiding principles in the process of selecting an approach and ultimately a final model. In our example, we did this by adopting the logarithmic model over a piecewise linear model, since the gain in fit was negligible relative to the increased complexity. This specific functional form may not be appropriate in other disease or therapeutic areas; careful analysis is required in each case to determine the best fit. Ultimately, selecting a “best fitting” model remains somewhat subjective. There is no single standard by which to judge “best”, and goodness-of-fit statistics may yield ambiguous or contradictory results. The choice of the model must be defensible using statistical and substantive considerations such as model complexity and interpretability.

The selection of an appropriate data source for PD costs is an important consideration. Where possible, a population that is comparable to that of the trial to which the results will apply should be used. Some compromise is likely to be required as only few sources may be available, and even then patient profiles may differ somewhat. Restricting or matching the populations may be useful, but should not be done at the cost of significant loss in data. In our example, we identified patients in the MA database with similar complications as those in ECLIPSE, and we truncated the LOS distributions to match the trials. This was done mainly for precautionary reasons, to avoid or minimize the impact of potential outliners. Relatively few observations were lost.

Some limitations of our analyses should be noted. For the estimation of pre-discharge costs by type of complication using the MA database, we know that the procedure was performed at that hospital admission, but we do not know if the events considered to be complications were present before or after the procedure. The complication group that was a mixture of ‘no complication’ patients with ‘other complication’ patients is a large, heterogeneous group. The nature of the ‘other’ complications is not considered in this analysis. We assumed that a common model was appropriate across complication groups. This was based on visual inspection of the scatter plots, and was not tested formally. We had no substantive clinical or economic reasons to believe otherwise, and the small size of some complication groups precluded the possibility of fitting separate models. We used regression terms for complication group and an interaction between this and LOS to allow some flexibility in the shape of the relationship across groups. Most of these terms were not statistically significant, however. This is likely due to collinearity between LOS and complication groups, as occurrence of the latter will lead to an increase of the former. The small size of some groups also limits the power to detect some of these interactions. We chose not to simplify the model by collapsing these complication groups because costs varied among different complications, and we wanted the PD cost model to be representative of the principles on which our exploratory analysis was based - that total cost should be a function of the LOS of the hospitalization, and should also appropriately reflect major complications that occurred.

PD costs are not independent of LOS in the MA Case Mix Database for those patients undergoing cardiac surgery. Fit statistics and substantive contextual considerations were used to select a suitable model of the relationship between PD cost and LOS in the MA Database to assign total costs to the ECLIPSE patients. This model had a logarithmic shape, which differs substantially from the horizontal shape used in the traditional/crude approach. The two models led to considerably different results in analyses of cost savings associated with “good” versus “poor” BP control.

Unbiased cost estimates are necessary to ascertain the true potential cost savings of therapeutic interventions such as BP control. Model-based methods capture dependence of PD cost on LOS, thus mitigating bias inherent in the traditional method of cost and cost savings estimation.

## Conclusions

PD costs are not independent of LOS in the example presented in this paper. This is likely to be the case in most situations where LOS can vary considerably across hospitalizations. The shape of the relationship between PD cost and LOS should not be assumed to be linear. The process described in this paper can be used to examine the shape of the relationship and model it with flexible techniques.

Unbiased cost estimates are necessary to ascertain the true potential cost savings of interventions such as BP control. Model-based methods capture dependence of PD cost on LOS, thus mitigating bias inherent in the traditional method of cost and cost savings estimation.

## Endnotes

^a^DRGs 104 through 109, 545 and 546 (2005–2006 data). The description labels for these DRGs are: DRG 104 cardiac valve and other major cardiothoracic procedures with cardiac catheterization; DRG 105 cardiac valve and other major cardiothoracic procedures without cardiac catheterization; DRG 106 coronary bypass with PTCA; DRG 107 coronary bypass with cardiac catheterization; DRG 108 other cardiothoracic procedures without congenital anomaly; DRG 109 coronary bypass without cardiac catheterization; DRG 545 cardiac valve procedure with major complications; and DRG 546 coronary bypass with major complications. For 2007, selected data also included DRGs 547 through 550 that replaced DRGs 106 through 109 from the 2005–2006 version.

^b^This value is 10% of the reported cost $20,906.23 for post-discharge infection; we assumed that only a small proportion would require re-hospitalization.

## Abbreviations

LOS: Length of stay; PD: Per diem; BP: Blood pressure; ECLIPSE: Evaluation of CLevidipine in the Perioperative Treatment of Hypertension Assessing Safety Events; HCUP: Healthcare Utilization Project; LOESS: LOcally wEighted Scatterplot Smoothing; AIC: Akaike information criterion; BIC: Bayesian information criterion.

## Competing interests

JI, MS, and DG are employed by the United BioSource Corporation (UBC), which provides consulting and other research services to pharmaceutical, device, government and non-government organizations. In this salaried position, JI, MS, and DG work with a variety of companies and organizations. They receive no payment or honoraria directly from these organizations for services rendered.

M-YH, YW, and GW are employees of The Medicines Company.

## Authors' contributions

All authors contributed equally to this work. All authors read and approved the final manuscript.

## Pre-publication history

The pre-publication history for this paper can be accessed here:

http://www.biomedcentral.com/1472-6963/12/439/prepub

## Supplementary Material

Additional file 1 **Appendix A.** ICD-9 CODES for ECLIPSE Complications. Click here for file
