# Supplementary material for: Accounting for the relationship between per diem cost and LOS when estimating hospitalization costs
Source: BMC Health Serv Res. 2012 Dec 1;12:439. doi: 10.1186/1472-6963-12-439 (PMC3522016; doi:10.1186/1472-6963-12-439)
Supplement: Additional file 1 — Appendix A. ICD-9 CODES for ECLIPSE Complications. [file 1472-6963-12-439-S1.docx]

**ICD-9 CODES for ECLIPSE Complications**

| Complication | icd_code | icd_desc |
| --- | --- | --- |
| Bleeding | 459.0 | UNSPECIFIED HEMORRHAGE |
| Bleeding | 531.00 | ACUT GASTR ULCR W/HEM W/O OBST |
| Bleeding | 531.01 | ACUT GASTR ULCER W/HEMORR&OBST |
| Bleeding | 531.20 | AC GASTR ULCR W/HEMOR&PERF W/O |
| Bleeding | 532.00 | ACUT DUOD ULCR W/HEM W/O OBST |
| Bleeding | 532.01 | ACUT DUOD ULCER W/HEMORR&OBST |
| Bleeding | 532.20 | AC DUOD ULCR W/HEMOR&PERF W/O |
| Bleeding | 532.21 | ACUT DUOD ULCER W/HEMORR PERF& |
| Bleeding | 533.00 | ACUT PEPTC ULCR W/HEM W/O OBST |
| Bleeding | 533.20 | ACUT PEP ULCR W/HEMOR&PERF W/O |
| Bleeding | 533.21 | ACUT PEPTC ULCR W/HEM PERF&OBS |
| Bleeding | 534.00 | ACUT GASTROJEJ ULCR W/HEM W/O |
| Bleeding | 534.01 | ACUT GASTROJEJUN ULCR W/HEMORR |
| Bleeding | 534.20 | ACUTE GASTROJEJ ULCR W/HEMORR& |
| Bleeding | 535.01 | ACUTE GASTRITIS WITH HEMORRHAG |
| Bleeding | 535.11 | ATROPHIC GASTRITIS WITH HEMORR |
| Bleeding | 535.41 | OTHER SPEC GASTRITIS W/HEMORRH |
| Bleeding | 578 | GASTROINTESTINAL HEMORR* |
| Bleeding | 578.0 | HEMATEMESIS |
| Bleeding | 578.9 | UNSPEC HEMORRHAGE GI TRACT |
| Bleeding | 599.7 | HEMATURIA |
| Bleeding | 784.7 | EPISTAXIS |
| Bleeding | 784.8 | HEMORRHAGE FROM THROAT |
| Bleeding | 786.3 | HEMOPTYSIS |
| Bleeding | 790.01 | PRECIPITOUS DROP IN HEMATOCRIT |
| Bleeding | 998.1 | HEMORR COMPLIC PROCEDURE |
| Bleeding | 998.11 | HEMORRHAGE COMPLICATING A PROC |
| Bleeding | 998.12 | HEMATOMA COMPLICATING A PROC N |
| Bleeding | 998.13 | SEROMA COMPLICATING A PROC NEC |
| Infection | 790.7 | BACTEREMIA |
| Infection | 996.6 | INFEC/INFLAM-DEVIC/GRAFT |
| Infection | 996.60 | INF&INFLAM REACT-UNS DEVC IMPL |
| Infection | 996.61 | INF&INFLAM REACT-CARD DEVC IMP |
| Infection | 996.62 | INF&INFLAM REACT TO VASC DEVIC |
| Infection | 996.69 | INF&INFLAM REACT INT PROS DEVI |
| Infection | 998.2 | ACC PUNCT/LACRATION DURING PRO |
| Infection | 998.3 | DISRUPTION OF OPERATION WOUND |
| Infection | 998.31 | DISRUPTION INTERNAL OPERATION |
| Infection | 998.32 | DISRUPTION EXTERNAL OPERATION |
| Infection | 998.5 | POSTOPERATIVE INFECTION* |
| Infection | 998.51 | INFECTED POSTOPERATIVE SEROMA |
| Infection | 998.59 | OTHER POSTOPERATIVE INFECTION |
| Infection | 998.83 | NON-HEALING SURGICAL WOUND NEC |
| Infection | 999.3 | OTH INFECTION DUE MEDICAL CARE |
| MI | 410.0 | AMI ANTEROLATERAL WALL* |
| MI | 410.00 | AC MI ANTEROLAT WALL EPIS CARE |
| MI | 410.01 | AC MI ANTEROLAT WALL INIT EPIS |
| MI | 410.1 | AMI ANTERIOR WALL NEC* |
| MI | 410.10 | ACUT MI OTH ANT WALL EPIS CARE |
| MI | 410.11 | ACUT MI OTH ANT WALL INIT EPIS |
| MI | 410.2 | AMI INFEROLATERAL WALL* |
| MI | 410.20 | AC MI INFEROLAT WALL EPIS CARE |
| MI | 410.21 | AC MI INFEROLAT WALL INIT EPIS |
| MI | 410.3 | AMI INFEROPOSTERIOR WALL |
| MI | 410.30 | AC MI INFEROPOST WALL EPIS CAR |
| MI | 410.31 | AC MI INFEROPOST WALL INIT EOC |
| MI | 410.4 | AMI INFERIOR WALL NEC* |
| MI | 410.40 | ACUT MI OTH INF WALL EPIS CARE |
| MI | 410.41 | ACUT MI OTH INF WALL INIT EPIS |
| MI | 410.5 | AMI LATERAL WALL NEC* |
| MI | 410.50 | ACUT MI OTH LAT WALL EPIS CARE |
| MI | 410.51 | ACUT MI OTH LAT WALL INIT EPIS |
| MI | 410.60 | ACUT MI POST WALL INFARCT EOC |
| MI | 410.61 | ACUT MI POST WALL INFARCT INIT |
| MI | 410.7 | SUBENDOCARDIAL INFARCT* |
| MI | 410.70 | ACUT MI SUBNDOCARDL INFARCT EO |
| MI | 410.71 | ACUT MI SUBNDOCRDL INFARCT INI |
| MI | 410.8 | MYOCARDIAL INFARCT NEC* |
| MI | 410.80 | ACUT MI OTH SITE EPIS CARE UNS |
| MI | 410.81 | ACUT MI OTH SITE INIT EPIS CAR |
| MI | 410.9 | MYOCARDIAL INFARCT NOS* |
| MI | 410.90 | ACUT MYOCARD INFARCT EPIS CARE |
| MI | 410.91 | ACUT MYOCARD INFARCT INIT EPIS |
| Renal dysfunction | 584.5 | ACUT RENL FAIL W/LES TUBULAR N |
| Renal dysfunction | 584.6 | AC RENL FAIL W/LES RENL CORT N |
| Renal dysfunction | 584.7 | AC RENL FAIL W/LES RENL MDLRY |
| Renal dysfunction | 584.8 | AC RENL FAIL W/OTH PATHAL LES |
| Renal dysfunction | 584.9 | UNSPECIFIED ACUTE RENAL FAILUR |
| Renal dysfunction | 586 | UNSPECIFIED RENAL FAILURE |
| Stroke | 430 | SUBARACHNOID HEMORRHAGE |
| Stroke | 431 | INTRACEREBRAL HEMORRHAGE |
| Stroke | 432.0 | NONTRAUMATIC EXTRADURAL HEMORR |
| Stroke | 432.1 | SUBDURAL HEMORRHAGE |
| Stroke | 432.9 | UNSPECIFIED INTRACRANIAL HEMOR |
| Stroke | 433.01 | OCCL&STENOS BASILAR ART W/INFA |
| Stroke | 433.11 | OCCL&STENOS CAROTID ART W/INFA |
| Stroke | 433.21 | OCCLUSION&STENOS VERT ART W/IN |
| Stroke | 433.31 | OCCL MX&BIL PRECERB ART-INFARC |
| Stroke | 433.81 | OCCL&STENOS OTH PRECERB ART-IN |
| Stroke | 433.91 | OCCL&STENOS UNS PRECERB ART-IN |
| Stroke | 434.00 | CERBRL THROMB W/O MENTION INFA |
| Stroke | 434.01 | CEREBRAL THROMBOSIS W/INFARCT |
| Stroke | 434.11 | CEREBRAL EMBOLISM W/INFARCT |
| Stroke | 434.91 | UNSPEC CERBRL ART OCCL W/INFAR |
| Stroke | 997.01 | CNS COMPLICATION NEC |
| Stroke | 997.02 | IATROGN CERBROVASC INFRCT/HEMR |
| Stroke | 997.09 | OTH NERVOUS SYS COMPLICATIONS |
